# Development and validation of risk prediction models for high-risk patients with non-traumatic acute abdominal pain: a prospective observational study

**DOI:** 10.3389/fpubh.2025.1701050

**Published:** 2025-12-09

**Authors:** Rui Li, Xiao-Hui Wei, Xiao-Qin Li, Ai-Hua Dong, Dan-Nan Ai, Li-Jin Zhou, Yan Yang

**Affiliations:** 1 School of Nursing, Shanghai Jiao Tong University, Shanghai, China; 2 Nursing Department, Shanghai Pulmonary Hospital, Shanghai, China; 3 Emergency Department, The First Affiliated Hospital of Soochow University, Suzhou, Jiangsu, China; 4 Tongren Hospital, School of Medicine, Shanghai Jiao Tong University, Shanghai, China; 5 Renji Hospital, Shanghai Jiao Tong University School of Medicine, Shanghai, China

**Keywords:** triage, abdominal pain, prediction model, machine learning, emergency nursing

## Abstract

**Purpose:**

This study develops and validates a machine learning–based model to help triage nurses identify high-risk patients with non-traumatic acute abdominal pain, enhancing accuracy and safety.

**Patients and methods:**

Utilizing information technology, a data collection form was embedded into the electronic pre-triage systems of the emergency departments in two tertiary general hospitals (Shanghai Tongren Hospital and the First Affiliated People’s Hospital of Soochow University). Data from 3,090 patients were prospectively collected and preprocessed. Predictive factors for non-traumatic acute abdominal pain were screened through univariate analysis, LASSO regression, and multivariate analysis. Risk early warning models were constructed using seven methods based on R software and externally validated at different time points.

**Results:**

The incidence of high-risk patients with non-traumatic acute abdominal pain was 14.49%. Ten predictive factors were identified: (1) age, (2) mode of admission, (3) history of heart disease, (4) history of tumor, (5) MEWS score ≥5, (6) trigger being post-coital, (7) knife-like pain, (8) accompanied by abdominal distension and fullness, (9) tenderness, and (10) muscle tension. All seven predictive models demonstrated good predictive performance, among which the random forest model (AUC = 0.786) showed the best overall predictive performance. External validation results indicated that the logistic regression model had good extrapolation and generalization ability. In this study, the logistic regression model was visualized using a nomogram.

**Conclusion:**

Machine learning models were developed for early risk prediction in non-traumatic acute abdominal pain; random forest showed the best discrimination, while logistic regression with a nomogram offered superior clinical applicability.

## 1 Introduction

Nontraumatic acute abdominal pain (NAAP) refers to acute abdominal pain caused by intra-abdominal, abdominal wall, thoracic, or systemic diseases, with an onset duration of less than 1 week, and may require emergency interventions such as surgery (1). Surveys have shown (2, 3) that nontraumatic acute abdominal pain accounts for as much as 5–10% of emergency department visits. The etiology of nontraumatic acute abdominal pain ranges from mild symptoms that do not require special treatment to severe conditions that necessitate emergency surgery or resuscitation (4, 5), such as gastroenteritis, urinary system calculi, acute appendicitis, intestinal obstruction, and gastrointestinal perforation. These conditions often involve multiple organs and systems (6, 7), posing significant challenges to clinical assessment and triage.

As the first step in the emergency diagnosis and treatment process, the quality of triage assessment directly determines the efficiency of subsequent medical procedures and the clinical outcomes of patients (8). In triage practice, triage errors mainly manifest in three types: undertriage and overtriage, classified based on the degree of deviation in assessment results, and misclassification errors caused by incorrect specialty judgment. Among these, undertriage is the most concerning due to its potential high risk. It specifically refers to the misjudgment of high-risk patients who require urgent care as being in a low-risk state, leading to delayed treatment and resulting in disease progression or even life-threatening consequences (9). Previous studies have shown (10) that the undertriage rate among patients with nontraumatic acute abdominal pain is as high as 31.0%, increasing the risks of delayed diagnosis and treatment, prolonged surgical time, and higher morbidity and mortality (11). Currently, there are few studies on predictive models for high-risk patients with nontraumatic acute abdominal pain, and existing studies have limitations (12, 13): they rely excessively on blood test indicators and imaging examinations, which are not applicable at the triage window; most are based on retrospective data, with a risk of selection bias in variable inclusion.

This study aims to develop and validate a high-risk early warning model for patients with non-traumatic acute abdominal pain using machine learning methods, in order to assist triage nurses in the early identification of high-risk patients with non-traumatic acute abdominal pain, improve triage accuracy, and optimize emergency resources.

## 2 Subjects and methods

### 2.1 Study subjects

This prospective observational study employed a convenience sampling method. Patients with non-traumatic acute abdominal pain who visited the emergency departments of two centers (Tongren Hospital and the First Affiliated People’s Hospital of Soochow University) with a total of three campuses between May 2024 and December 2024 were included as study subjects. These institutions represent two medical centers and three hospital districts. Inclusion criteria included the following: (1) Age ≥ 16 years; (2) Diagnosis of non-traumatic acute abdominal pain based on the “Guidelines for the Primary Care of Acute Abdominal Pain 2015” (1); (3) Follow-up visits. Exclusion criteria were as follows: (1) History of abdominal trauma, surgery, or other invasive abdominal procedures within the past 30 days; (2) Pregnant individuals; (3) Incomplete medical records.

The sample size was calculated using the predictive model sample size estimation method recommended by Riley et al. (14): N = (1.96/*δ*)^2^ × *p* (1−*p*), where δ (set at 3%) denotes the allowable error margin for high-risk non-traumatic acute abdominal pain outcomes, and *p* (26%) represents the incidence of high-risk cases. Consequently, the required sample size was determined as follows: N = (1.96/0.03)^2^ × 0.26 (1–0.26) = 820. Adjusting for a 10–15% potential loss to follow-up and recognizing that larger sample sizes improve the predictive model’s accuracy, the final sample size was set at 3090 cases. Among them, 2,471 cases collected from May to October 2024 were used for model training, while 619 cases collected from November to December 2024 were used for external validation across time periods. This study was approved by the ethics committees of two hospitals in Shanghai (Protocol No: 2024-001-01) and Suzhou (Protocol No: 2024-720), and written informed consent was obtained from all participants. Written informed consent was obtained from all participants. The study was carried out in accordance with the applicable guidelines and regulations.

### 2.2 Investigative tools

#### 2.2.1 Data collection form

A data collection form was developed based on a comprehensive literature review, expert consultations, and clinical practice considerations to identify the characteristic variables associated with high-risk factors for non-traumatic acute abdominal pain.

The form contains four sections as follows:

1) General Characteristics (13 items): age, gender, mode of admission [ambulance arrival (any emergency medical-service transport, including EMS and hospital-based ambulances) versus non-ambulance arrival (walk-in, private vehicle, public transport, etc.)], the time interval between the last meal and seeking medical attention, the number of underlying conditions, past medical history, reproductive history, menstrual history, the presence of vaginal bleeding, menstrual flow volume, history of abdominal surgery, allergy history, and medication history.
2) Characteristics of Abdominal Pain (6 items): duration of pain, triggering factors, nature of pain, pain location, associated symptoms, and pain frequency.
3) Positive Signs (5 items): tenderness, rebound tenderness, muscle tension, hepatic percussion tenderness, and renal percussion tenderness.
4) Vital Signs and Scores (8 items): body temperature, oxygen saturation (SPO2), Modified Early Warning Score [MEWS (15)], heart rate, systolic blood pressure, diastolic blood pressure, Numerical Rating Scale (NRS) score, and shock index.

#### 2.2.2 Classification criteria for high- and non-high risk non-traumatic acute abdominal pain

The causes of acute abdominal pain can be categorized into high-risk and low-risk groups. This study adopted the definition of high-risk patients from the OPTIMA study (16), which describes them as: “cases requiring medical intervention within 24 h to prevent severe complications.” This definition has also been used by other researchers (17–19). The criteria for high-risk classification include non-traumatic acute abdominal pain patients who require medical intervention and cannot be discharged within 24 h. These circumstances include emergency observation (≥24 h), hospital admission, emergency interventional procedures or surgery, death, and atypical discharge events, where atypical discharge specifically refers to patients who leave the hospital against medical advice. Discharge against medical advice refers to patients leaving the hospital without following the medical advice given (20). During the process of receiving medical treatment, the discharge decision is not made by the doctor based on an assessment of the patient’s condition; instead, the patient or their family chooses to prematurely terminate treatment and leave the hospital or transfer to another facility for personal reasons before the diagnostic and treatment process is completed.

### 2.3 Data collection

After obtaining consent from the heads of relevant departments at the two selected hospitals, the researchers proceeded with data collection. The data collection was mainly divided into three stages. In the first stage, the researchers coordinated and communicated with information engineers from three hospital campuses, embedding the risk factor questionnaire into the emergency electronic pre-triage system through information technology, and provided standardized training to all general nurses. In the second stage, pre-triage nurses conducted interviews and recorded information for non-traumatic acute abdominal pain patients who met the inclusion and exclusion criteria. In the third stage, engineers imported the collected data into Excel software, and the researchers then used the patients’ visit ID numbers to retrieve and complete information such as emergency department outcomes.

### 2.4 Data preparation and handling of missing values

Research team members from the three hospital campuses (one graduate student and one clinical consultant each) conducted three rounds of data screening and cleaning. The first round involved excluding duplicate records and entries with missing data. The second round strictly followed the inclusion and exclusion criteria to further screen the database. In the third round, records with questionable numerical values were reviewed. Based on the clinical consultant’s domain knowledge (e.g., oxygen saturation greater than 100%), observations with excessive missing data, duplicate entries, and erroneous vital signs were removed to ensure the accuracy of the database. A total of 3,090 valid patient records were ultimately included. These records were then uniformly coded by two graduate students.

For missing data imputation, the “mice” package in the R software was employed. The random forest algorithm was used to perform 5-fold cross-validation for imputing missing values in the dataset. The dataset with the optimal imputation results was selected for subsequent analyses, ensuring both accuracy and reliability.

### 2.5 Statistical analysis and model construction

#### 2.5.1 Statistical analysis

Data analysis was conducted using the IBM SPSS 27.0 and R 4.4.0 statistical software. Categorical data were expressed as frequencies and percentages (%), while continuous data were presented as means ± standard deviations, medians, or interquartile ranges. Missing values were addressed through regression-based imputation methods. For univariate analysis, the chi-square test or Fisher’s exact test was utilized for categorical data, and the non-parametric Mann–Whitney test was adopted for continuous data. A *p*-value of less than 0.05 was considered statistically significant.

#### 2.5.2 Model construction

**Figure 1:**
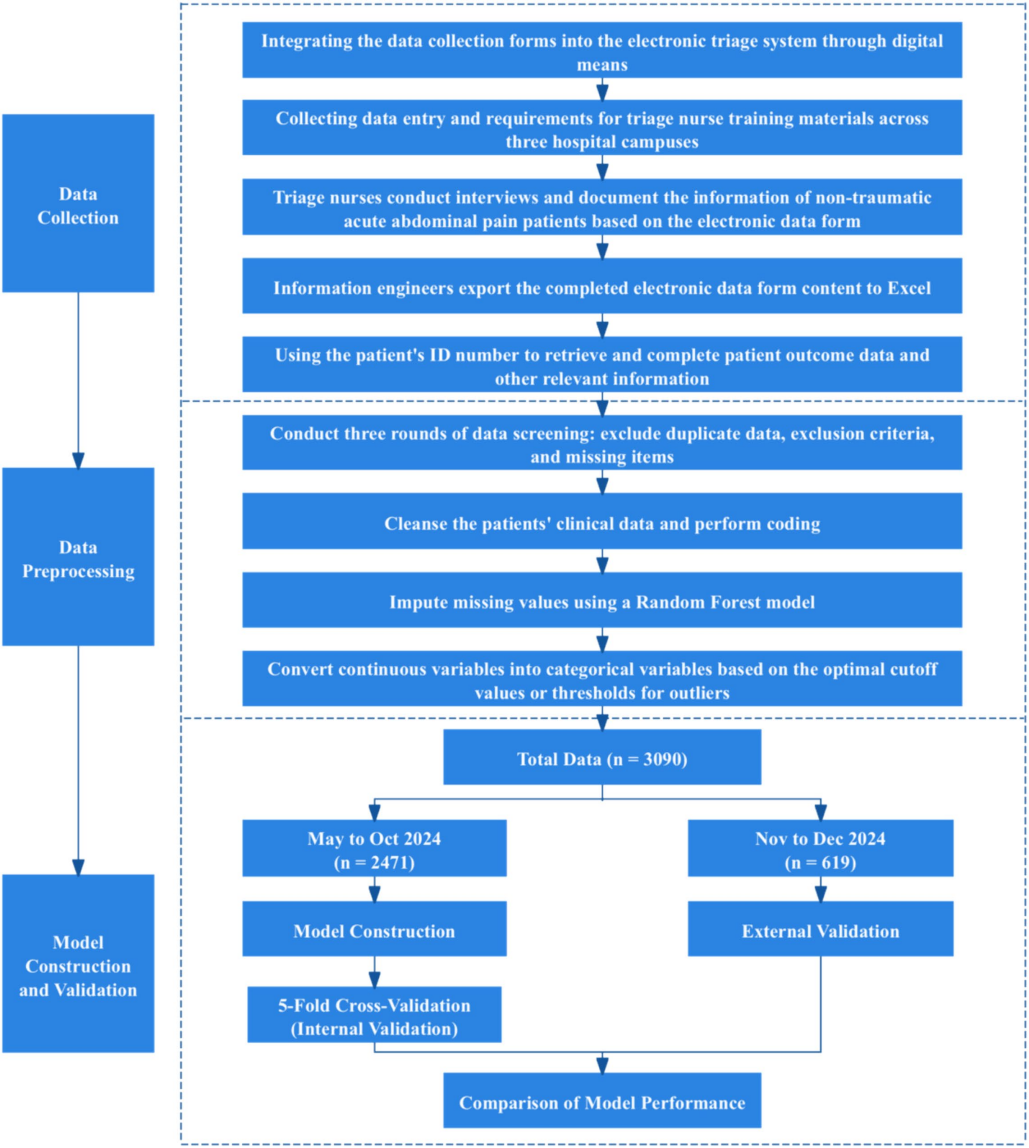
Model development flow chart.

Patient data collected between May 2024 and October 2024 were used for model construction, while data from November 2024 to December 2024 were employed for external validation across different time periods. The model development flow chart is presented in Figure 1. A five-fold cross-validation method was implemented for the internal validation of the model. The detailed methodology is as follows:

##### 2.5.2.1 Risk factor screening

In this study, univariate analysis, LASSO regression, and multivariate analysis were employed to screen the risk factors most relevant to the target variable for model training. The LASSO regression algorithm is an estimation method capable of simplifying the set of indicators (21), effectively addressing the issue of multicollinearity among variables, thereby avoiding model overfitting, improving the accuracy of model estimation, and enhancing the evaluation and application of the model (22, 23). The specific procedure was as follows: variables with statistical significance in univariate analysis were included in the LASSO regression, and ten-fold cross-validation was used to select the optimal variables. Subsequently, the variables selected by LASSO regression were incorporated into the logistic regression model, and those with statistical significance in the logistic regression model were used as the final selected risk factors for model construction.

##### 2.5.2.2 Model training and hyperparameter tuning

This study constructed an early warning model based on seven algorithms, including logistic regression, k-nearest neighbors (KNN), Light Gradient Boosting Machine (LightGBM), neural networks, Extreme Gradient Boosting (XGBoost), random forest, and support vector machine (SVM). Data from May to October 2024 were used as the training set for model training, and five-fold cross-validation was employed for hyperparameter tuning. Model training and hyperparameter grid search were conducted using the “workflows” and “tune” packages in R software.

##### 2.5.2.3 Model evaluation

Model performance was evaluated using various metrics, including the area under the ROC curve (AUC), accuracy, precision, recall, and F1 score, The clinical applicability of the model was evaluated through Decision Curve Analysis (DCA).

##### 2.5.2.4 External validation

This study employed a temporal validation framework system to externally validate seven early warning models. Specifically, data from November to December 2024 were used as the test set to evaluate the models’ generalization ability across different time periods.

##### 2.5.2.5 Model visualization

To improve the clinical utility of the model, the logistic regression results were visualized using a nomogram.

## 3 Results

### 3.1 Comparison of baseline characteristics between training and validation cohorts

A total of 3,090 non-traumatic acute abdominal pain patients meeting the inclusion and exclusion criteria were enrolled in this study. The training group included 2,471 patients, among whom 358 cases (14.49%) experienced high-risk outcomes; the validation group included 619 patients, among whom 90 cases (14.54%) experienced high-risk outcomes. A detailed comparison of baseline characteristics between the training and validation groups is presented in Table 1.

**Table 1:** Comparison of baseline characteristics between training and validation set.

Variables	Training set (*n* = 2,471)	Validation set (*n* = 619)	Statistic	*p*
High risk			*χ*^2^ = 0.00	0.974
No	2,113 (85.51)	529 (85.46)		
Yes	358 (14.49)	90 (14.54)		
Age	42.00 (30.00, 58.00)	41.00 (30.00, 59.00)	*Z* = −0.27	0.790
Time of last meal (hours)			*χ*^2^ = 0.00	0.959
<7.5	1,606 (64.99)	403 (65.11)		
≥7.5	865 (35.01)	216 (34.89)		
Gender			*χ*^2^ = 2.77	0.096
Male	1,162 (47.03)	268 (43.30)		
Female	1,309 (52.97)	351 (56.70)		
Mode of admission			*χ*^2^ = 2.39	0.122
Non-ambulance Admission	2,360 (95.51)	582 (94.02)		
Ambulance admission	111 (4.49)	37 (5.98)		
Number of comorbidities			*χ*^2^ = 0.02	0.889
≤1	2,386 (96.56)	597 (96.45)		
≥2	85 (3.44)	22 (3.55)		
Hypertension history	205 (8.30)	47 (7.59)	*χ*^2^ = 0.33	0.567
Diabetes history	91 (3.68)	22 (3.55)	*χ*^2^ = 0.02	0.879
Cardiovascular disease history	44 (1.78)	8 (1.29)	*χ*^2^ = 0.71	0.398
Cerebrovascular disease history	9 (0.36)	3 (0.48)	*χ*^2^ = 0.00	0.945
Hyperlipidemia history	10 (0.40)	2 (0.32)	*χ*^2^ = 0.00	1.000
Tumor history	71 (2.87)	11 (1.78)	*χ*^2^ = 2.30	0.129
Hepatic disease history	13 (0.53)	2 (0.32)	*χ*^2^ = 0.11	0.744
Renal disease history	23 (0.93)	6 (0.97)	*χ*^2^ = 0.01	0.929
Biliary tract disease history	164 (6.64)	43 (6.95)	*χ*^2^ = 0.08	0.783
Pancreatitis history	30 (1.21)	6 (0.97)	*χ*^2^ = 0.26	0.612
Intestinal obstruction history	17 (0.69)	4 (0.65)	*χ*^2^ = 0.00	1.000
Abdominal surgery history	319 (12.91)	94 (15.19)	*χ*^2^ = 2.21	0.137
Allergy history	84 (3.40)	13 (2.10)	*χ*^2^ = 2.75	0.097
Medication history	551 (22.30)	153 (24.72)	*χ*^2^ = 1.65	0.200

Z, Mann–Whitney test; *χ*^2^, Chi-square test.

### 3.2 Univariate analysis of high-risk patients with non-traumatic acute abdominal pain

#### 3.2.1 Analysis of general characteristics of high-risk patients with non-traumatic acute abdominal pain

Univariate analysis revealed statistically significant differences (*p* < 0.05) between the high-risk and non-high-risk groups across 17 variables: age, mode of admission, number of underlying conditions, history of hypertension, diabetes, heart disease, cerebrovascular disease, tumors, gallbladder disease, pancreatitis, intestinal obstruction, reproductive history, menstrual history, vaginal bleeding, menstrual volume, allergy history, and time of last meal. Details are provided in Table 2.

**Table 2:** Analysis of general characteristics of high-risk patients with non-traumatic acute abdominal pain.

Variables	Non-high risk (*N* = 2,113) *n* (%)	High-risk (*N* = 358) *n* (%)	Statistic	*p*
Age	40.00 (29.00, 56.00)	53.00 (36.00, 69.00)	*Z* = −8.53	**<0.001**
Time of last meal (hours)			*χ*^2^ = 8.05	**0.005**
<7.5	1,397 (86.99)	209 (13.01)		
≥7.5	716 (82.77)	149 (17.23)		
Gender			*χ*^2^ = 1.49	0.223
Male	983 (84.60)	179 (15.40)		
Female	1,130 (86.33)	179 (13.67)		
Mode of admission			*χ*^2^ = 51.15	**<0.001**
Non-ambulance admission	2044 (86.61)	316 (13.39)		
Ambulance admission	69 (62.16)	42 (37.84)		
Number of comorbidities			*χ*^2^ = 34.34	**<0.001**
≤1	2059 (86.30)	327 (13.70)		
≥2	54 (63.53)	31 (36.47)		
Past medical history				
Hypertension	147 (71.71)	58 (28.29)	*χ*^2^ = 34.39	**<0.001**
Diabetes	64 (70.33)	27 (29.67)	*χ*^2^ = 17.58	**<0.001**
Cardiovascular disease	22 (50.00)	22 (50.00)	*χ*^2^ = 45.60	**<0.001**
Cerebrovascular disease	4 (44.44)	5 (55.56)	*χ*^2^ = 9.19	**0.002**
Hyperlipidemia	7 (70.00)	3 (30.00)	*χ*^2^ = 0.90	0.344
Tumor	48 (67.61)	23 (32.39)	*χ*^2^ = 18.92	**<0.001**
Hepatic disease	9 (69.23)	4 (30.77)	*χ*^2^ = 1.63	0.202
Renal disease	21 (91.30)	2 (8.70)	*χ*^2^ = 0.25	0.620
Biliary tract disease	123 (75.00)	41 (25.00)	*χ*^2^ = 15.67	**<0.001**
Pancreatitis	20 (66.67)	10 (33.33)	*χ*^2^ = 7.23	**0.007**
Intestinal obstruction	10 (58.82)	7 (41.18)	*χ*^2^ = 7.79	**0.005**
Obstetric history			*χ*^2^ = 14.27	**<0.001**
Male/postmenopausal	1,305 (83.49)	258 (16.51)		
No	704 (89.23)	85 (10.77)		
Yes	104 (87.39)	15 (12.61)		
Menstrual history			*χ*^2^ = 11.27	**0.010**
Male/postmenopausal	1,358 (83.88)	261 (16.12)		
Normal	691 (88.59)	89 (11.41)		
Delayed	54 (87.10)	8 (12.90)		
Advanced	10 (100.00)	0 (0.00)		
Menstrual volume			*χ*^2^ = 17.26	**<0.001**
Male/postmenopausal	1,342 (83.88)	258 (16.12)		
Normal	539 (90.74)	55 (9.26)		
Abnormal	232 (83.75)	45 (16.25)		
Vaginal bleeding	39 (97.50)	1 (2.50)	*χ*^2^ = 4.72	**0.030**
Abdominal surgery history	265 (83.07)	54 (16.93)	*χ*^2^ = 1.76	0.185
Allergy history	64 (76.19)	20 (23.81)	*χ*^2^ = 6.10	**0.014**
Medication history	481 (87.30)	70 (12.70)	*χ*^2^ = 1.82	0.177

Z, Mann–Whitney test; *χ*^2^, Chi-square test; −, Fisher exact; *, Simulated *p*-value. Bold type indicates *p* < 0.05.

#### 3.2.2 Analysis of abdominal characteristics in high-risk patients with non-traumatic acute abdominal pain

The univariate analysis revealed statistically significant differences (*p* < 0.05) between high-risk and non-high-risk groups in nine abdominal characteristics: pain duration, pain triggers, pain nature, pain location, and associated symptoms, including nausea, vomiting, diarrhea, back pain, and abdominal distension. Detailed findings are presented in Table 3.

**Table 3:** Analysis of abdominal characteristics in high-risk patients with non-traumatic acute abdominal pain.

Variables	Non-high risk (*N* = 2,113) *n* (%)	High-risk (*N* = 358) *n* (%)	Statistic	*p*
Duration of pain (hours)			*χ*^2^ = 13.06	**<0.001**
≤9	1,162 (87.90)	160 (12.10)		
≥9	951 (82.77)	198 (17.23)		
Pain frequency			*χ*^2^ = 0.49	0.484
Continuous	1,022 (86.03)	166 (13.97)		
Paroxysmal	1,091 (85.04)	192 (14.96)		
Pain triggers			-	<0.001*
None	1898 (86.59)	294 (13.41)		
After eating	141 (83.43)	28 (16.57)		
After alcohol consumption	3 (100.00)	0 (0.00)		
After fatty meal	17 (73.91)	6 (26.09)		
After unhealthy diet	22 (70.97)	9 (29.03)		
After sexual intercourse	17 (51.52)	16 (48.48)		
Other	15 (75.00)	5 (25.00)		
Pain characteristics			*χ*^2^ = 29.09	**<0.001**
Throbbing pain	73 (89.02)	9 (10.98)		
Stabbing pain	88 (83.02)	18 (16.98)		
Cutting	9 (52.94)	8 (47.06)		
Dull	116 (88.55)	15 (11.45)		
Colicky	699 (83.71)	136 (16.29)		
Burning	9 (90.00)	1 (10.00)		
Hidden	333 (91.23)	32 (8.77)		
Bloating	786 (84.97)	139 (15.03)		
Pain location			*χ*^2^ = 40.34	**<0.001**
Left lower abdomen	288 (91.72)	26 (8.28)		
Right upper abdomen	169 (83.66)	33 (16.34)		
Right lower abdomen	367 (82.84)	76 (17.16)		
Right mid abdomen	58 (92.06)	5 (7.94)		
Mid abdomen	355 (83.92)	68 (16.08)		
Lower mid abdomen	156 (75.73)	50 (24.27)		
Upper mid abdomen	459 (87.43)	66 (12.57)		
Left mid abdomen	52 (98.11)	1 (1.89)		
Left upper abdomen	104 (86.67)	16 (13.33)		
Generalized abdomen	105 (86.07)	17 (13.93)		
Simultaneous phenomenon				
Nausea	461 (81.59)	104 (18.41)	*χ*^2^ = 9.08	**0.003**
Vomiting	369 (81.10)	86 (18.90)	*χ*^2^ = 8.77	**0.003**
Diarrhea	250 (92.59)	20 (7.41)	*χ*^2^ = 12.27	**<0.001**
Flatulence	45 (78.95)	12 (21.05)	*χ*^2^ = 2.03	0.154
Constipation	22 (75.86)	7 (24.14)	*χ*^2^ = 1.49	0.223
Low back pain	172 (95.03)	9 (4.97)	*χ*^2^ = 14.27	**<0.001**
Fever	74 (87.06)	11 (12.94)	*χ*^2^ = 0.17	0.680
Abdominal distention	100 (72.46)	38 (27.54)	*χ*^2^ = 20.09	**<0.001**
Back pain	50 (79.37)	13 (20.63)	*χ*^2^ = 1.97	0.160
Chest pain	15 (75.00)	5 (25.00)	*χ*^2^ = 1.04	0.307
Hematochezia	5 (62.50)	3 (37.50)	*χ*^2^ = 1.82	0.177
Urinary tract irritation signs	22 (91.67)	2 (8.33)	*χ*^2^ = 0.32	0.569

Z, Mann–Whitney test; *χ*^2^, Chi-square test; −, Fisher exact; *, Simulated *p*-value. Bold type indicates *p* < 0.05.

#### 3.2.3 Analysis of abdominal signs in high-risk patients with non-traumatic acute abdominal pain

The results of the univariate analysis revealed statistically significant differences in five abdominal signs between the high-risk and non-high-risk groups: tenderness, rebound tenderness, muscular tension, liver percussion tenderness, and kidney percussion tenderness (*p* < 0.05). Detailed results are presented in Table 4.

**Table 4:** Analysis of abdominal features in high-risk patients with non-traumatic acute abdominal pain.

Variables	Non-high risk (*N* = 2,113) *n* (%)	High-risk (*N* = 358) *n* (%)	Statistic	*p*
Tenderness			χ^2^ = 128.18	**<0.001**
Absent	1,397 (91.85)	124 (8.15)		
Present	716 (75.37)	234 (24.63)		
Rebound tenderness			χ^2^ = 58.26	**<0.001**
Absent	2076 (86.43)	326 (13.57)		
Present	37 (53.62)	32 (46.38)		
Muscle rigidity			χ^2^ = 93.11	**<0.001**
Absent	2,112 (86.13)	340 (13.87)		
Present	1 (5.26)	18 (94.74)		
Hepatic percussion tenderness			χ^2^ = 6.23	**0.013**
Absent	2,107 (85.65)	353 (14.35)		
Present	6 (54.55)	5 (45.45)		
Renal percussion tenderness			χ^2^ = 7.01	**0.008**
Absent	1971 (85.03)	347 (14.97)		
Present	142 (92.81)	11 (7.19)		

Z, Mann–Whitney test; *χ*^2^, Chi-square test; −, Fisher exact; *, Simulated *p*-value. Bold type indicates *p* < 0.05.

#### 3.2.4 Analysis of vital signs and scores in non-traumatic acute abdominal pain

Univariate analysis results indicated statistically significant differences (*p* < 0.05) between high-risk and non-high-risk groups in terms of heart rate, diastolic blood pressure, SpO2, MEWS, and NRS. Detailed results are presented in Table 5.

**Table 5:** Analysis of vital signs and related scores in high-risk patients with non-traumatic acute abdominal pain.

Variables	Non-high risk (*N* = 2,113) *n* (%)	High-risk (*N* = 358) *n* (%)	Statistic	*p*
Body temperature	36.50 (36.50, 36.80)	36.50 (36.50, 36.60)	*Z* = −0.58	0.560
Heart rate	82.00 (73.00, 92.00)	84.50 (71.00, 98.00)	*Z* = −2.53	**0.011**
Systolic blood pressure	129.00 (116.00, 146.00)	131.00 (115.00, 149.00)	*Z* = −0.81	0.417
Diastolic blood pressure	79.00 (71.00, 88.00)	76.00 (68.00, 85.00)	*Z* = −3.74	**<0.001**
SPO2	97.00 (96.00, 99.00)	96.00 (96.00, 97.00)	*Z* = −9.23	**<0.001**
SI	0.63 (0.54, 0.75)	0.66 (0.53, 0.79)	*Z* = −1.50	0.133
Mews			*χ*^2^ = 54.77	**<0.001**
<5	2,108 (86.01)	343 (13.99)		
≥5	5 (25.00)	15 (75.00)		
NRS			*χ*^2^ = 20.38	**<0.001**
0–3	1,356 (87.71)	190 (12.29)		
4–6	728 (82.35)	156 (17.65)		
7–10	29 (70.73)	12 (29.27)		

Z, Mann–Whitney test; *χ*^2^, Chi-square test; −, Fisher exact; *, Simulated *p*-value. Bold type indicates *p* < 0.05.

### 3.3 LASSO regression analysis of high-risk patients with non-traumatic acute abdominal pain

**Figure 2:**
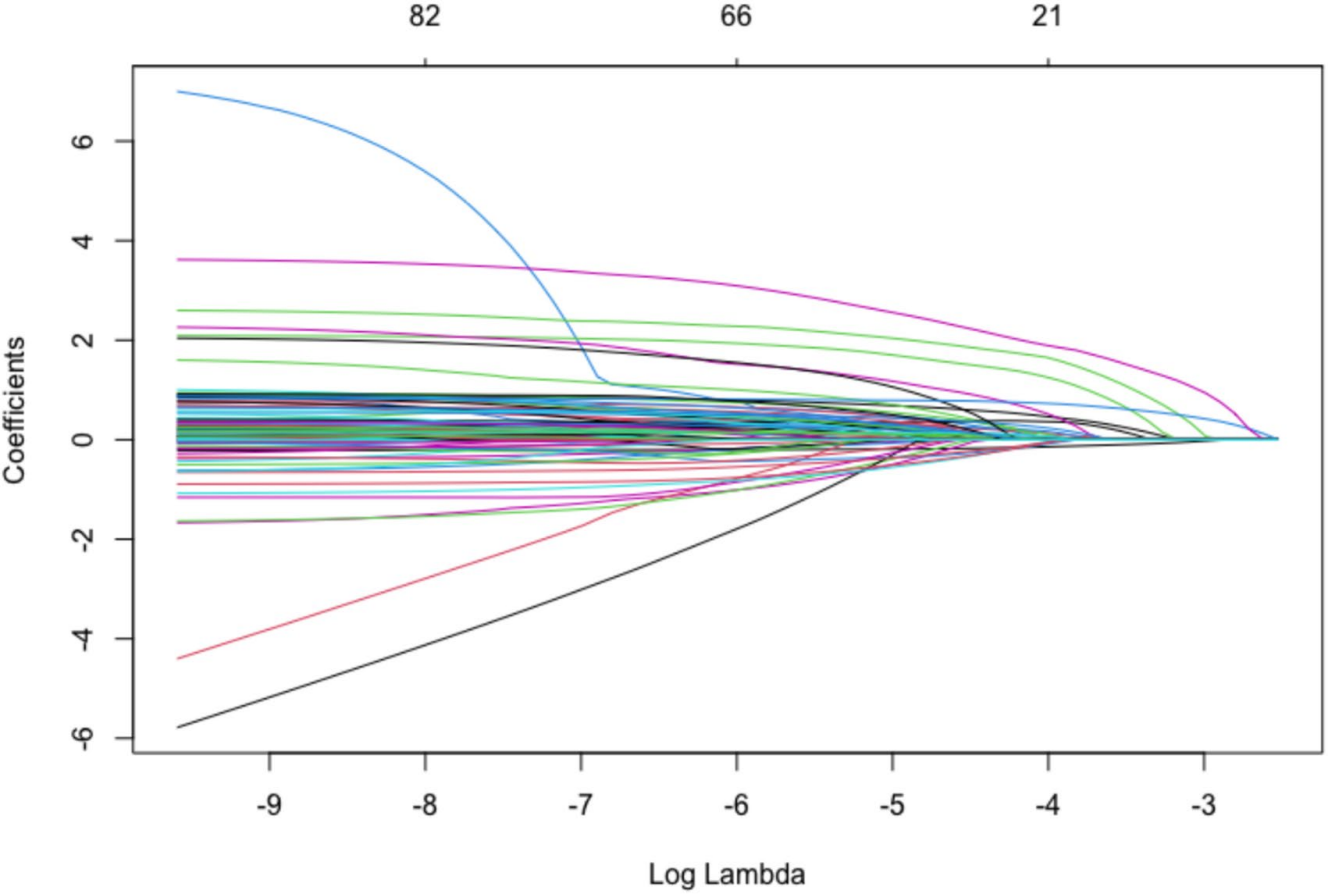
LASSO regression coefficients of variables.

**Figure 3:**
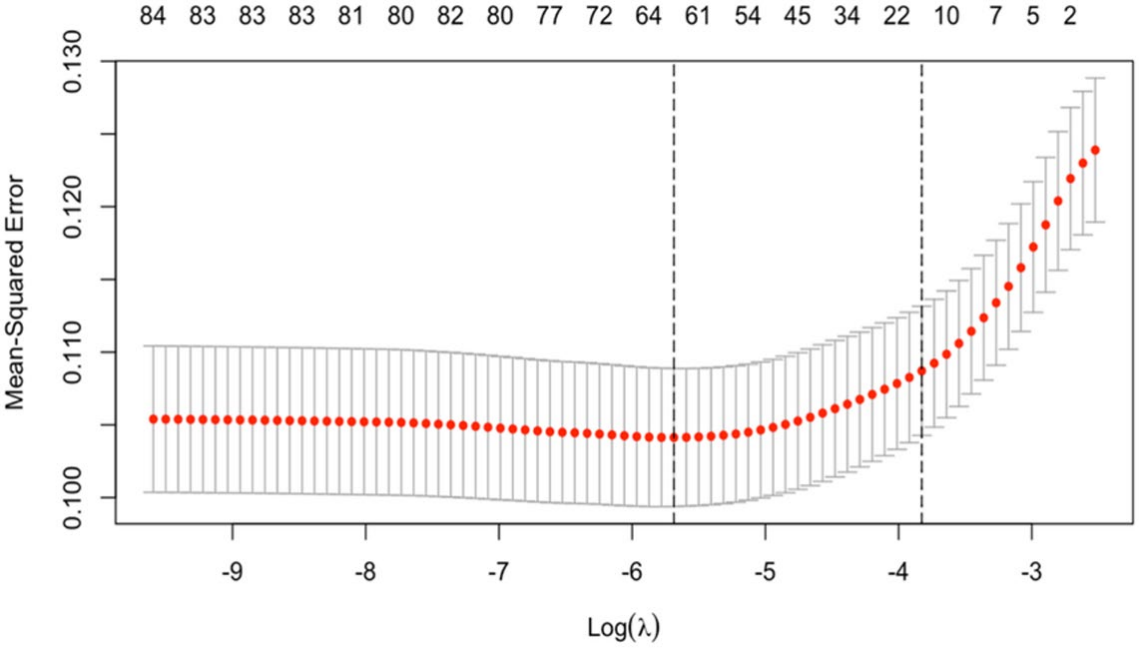
Results of LASSO 10-fold cross-validation.

A total of 36 significant variables identified from univariate analysis were included in the LASSO regression for screening, as shown in Figure 2. To make the model more concise and applicable for clinical use, we selected *λ* at lambda.1se as the optimal value. At this point, the model’s optimal variables numbered 11, including age, admission method, number of comorbidities, history of heart disease, history of cancer, MEWS, triggering factors, nature of pain, abdominal distension, tenderness, and muscle tension, as illustrated in Figure 3.

### 3.4 Multivariate logistic regression analysis of high-risk patients with non-traumatic acute abdominal pain

High-risk abdominal pain was used as the dependent variable, with 11 optimal variables identified through LASSO regression included as independent variables in the multivariate logistic regression analysis. Four modeling approaches were used for the analysis: overall regression (AIC = 1721.665), forward selection (AIC = 1721.665), backward elimination (AIC = 1719.955), and bidirectional regression (AIC = 1719.955). Among these, bidirectional logistic regression was identified as the optimal subset model. The study concluded that 10 variables were risk factors influencing the triaging of patients with non-traumatic acute abdominal pain: age, mode of admission, history of heart disease, history of tumors, MEWS ≥5, post-coital onset, knife-like pain, accompanying abdominal distension, tenderness, and muscle rigidity. Detailed results are available in Table 6.

**Table 6:** Multivariate logistic regression analysis of patients with acute non-traumatic high-risk abdominal pain.

Variables	*B*	SE	OR	CI	*Z*	*p*
Age	0.023	0.004	1.023	1.015–1.031	6.462	<0.001
Mode of admission	0.894	0.238	2.445	1.534–3.898	3.761	<0.001
History of heart disease	0.995	0.355	2.705	1.349–5.425	2.800	0.005
History of tumors	0.853	0.291	2.347	1.327–4.151	2.935	0.003
MEWS≥5	2.855	0.575	17.380	5.631–53.642	4.963	<0.001
Post-coital onset	2.416	0.378	11.197	5.338–23.49	6.390	<0.001
Cutting-like pain	1.749	0.542	5.751	1.988–16.637	3.227	0.001
Abdominal distension	0.729	0.226	2.073	1.331–3.229	3.225	0.001
Tenderness,	1.133	0.128	3.105	2.416–3.99	8.819	<0.001
Muscle rigidity	3.579	1.044	35.832	4.63–277.297	3.429	0.001

### 3.5 Construction and validation of a high-risk prediction model for patients with non-traumatic acute abdominal pain

This study employed seven different machine learning algorithms, including logistic regression, k-nearest neighbors (KNN), Light Gradient Boosting Machine (LightGBM), neural networks, random forest, support vector machine (SVM), and eXtreme Gradient Boosting (XGBoost), to construct and validate a high-risk early warning model for patients with non-traumatic acute abdominal pain. Each algorithm was trained using 5-fold cross-validation combined with hyperparameter grid search to enhance performance and mitigate overfitting. Additionally, the internal validation phase applied 5-fold cross-validation to evaluate the robustness and reliability of the models.

**Figure 4:**
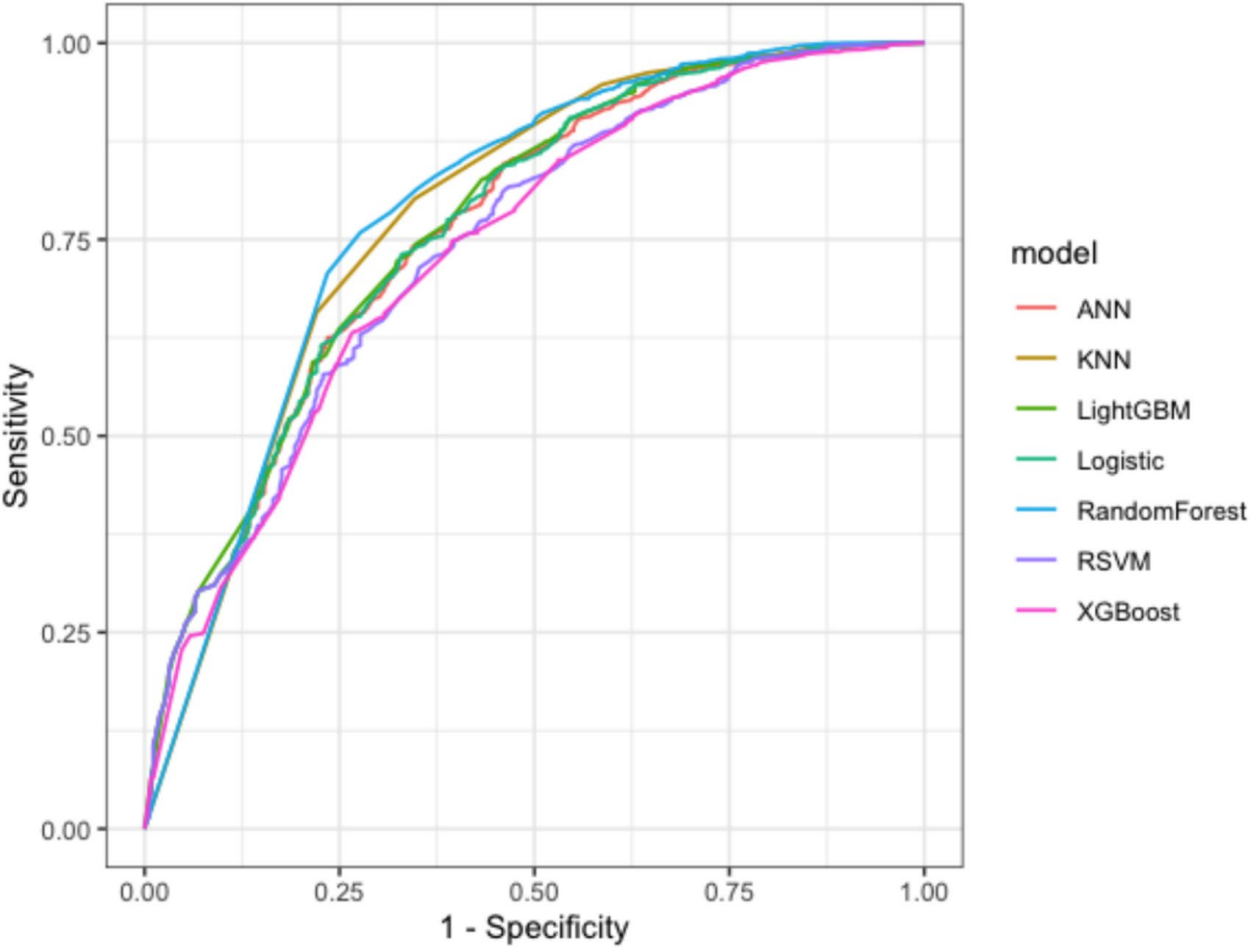
ROC curves of seven risk warning models.

This study provides a comparative analysis of accuracy, precision, recall, F1 score, and AUC values for seven predictive models. The results indicate that the Random Forest model exhibits a relatively high AUC value of 0.786 and achieves a precision rate of 0.942, demonstrating its strong classification ability in differentiating between high-risk patients with non-traumatic acute abdominal pain and those who are non-high-risk. The KNN model recorded a recall rate of 0.802, indicating higher accuracy in identifying high-risk patients and enhancing its ability to recognize positive samples. Furthermore, models such as Logistic Regression and Neural Networks demonstrated unique characteristics. Detailed results are presented in Table 7. By plotting ROC curves based on variations in AUC values and analyzing their overlaps, it was observed that all four models exhibited excellent predictive performance, with the ROC curve of the Random Forest model (blue curve) outperforming the others. Refer to Figure 4 for more details.

**Table 7:** Performance evaluation results for seven risk prediction models.

Models	Accuracy	Precision	Recall	F1	AUC
Logistic	0.722	0.929	0.731	0.818	0.765
KNN	0.780	0.932	0.802	0.862	0.780
LightGBM	0.716	0.929	0.723	0.813	0.769
ANN	0.728	0.927	0.741	0.823	0.763
RF	0.754	0.942	0.759	0.840	0.786
SVM	0.704	0.923	0.714	0.805	0.743
XGBoost	0.647	0.933	0.633	0.754	0.736

### 3.6 External validation results of the models

**Figure 5:**
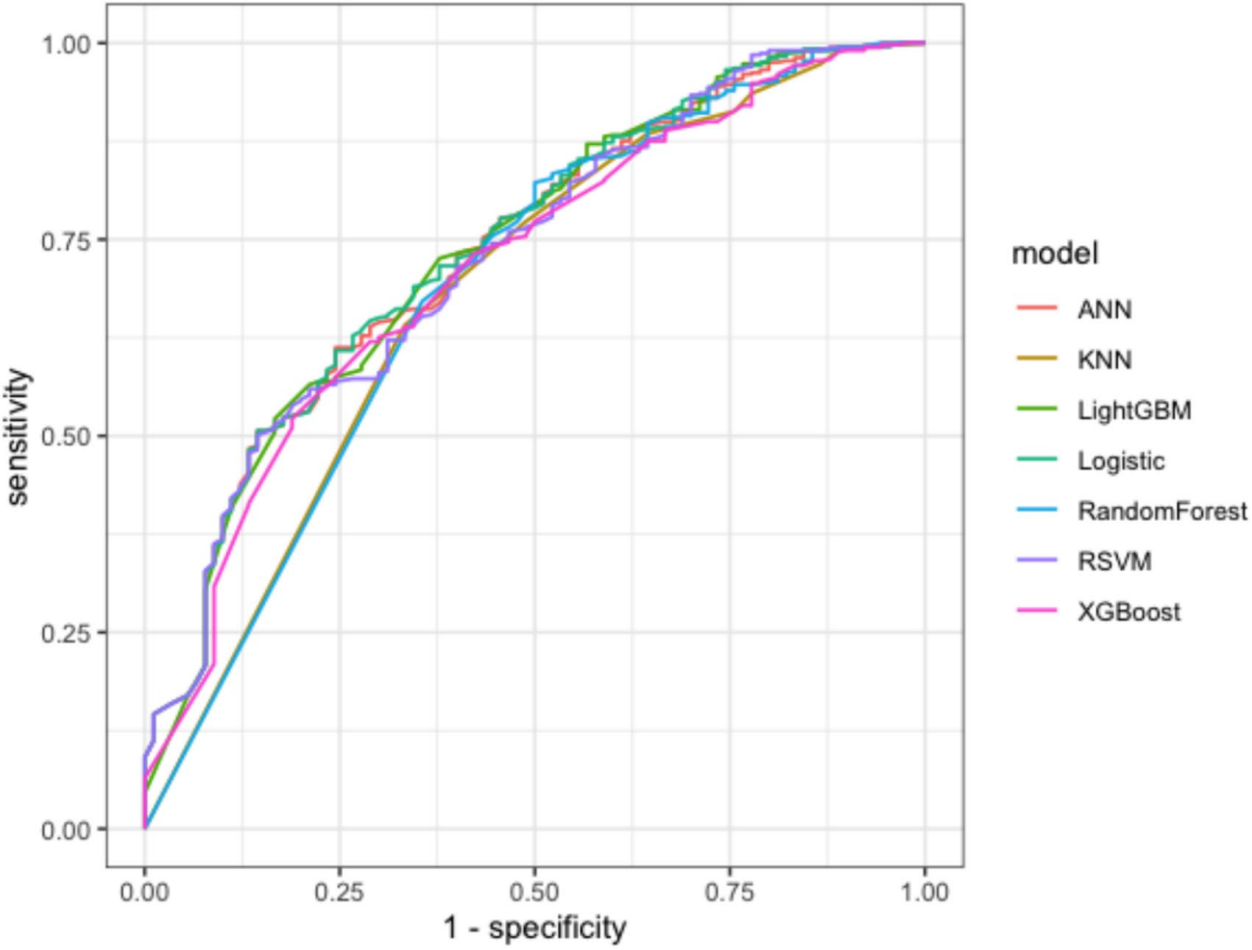
ROC curves for the external validation of the seven risk prediction models.

To further validate the generalization ability of the model, this study conducted external validation across different time periods. In the validation set evaluation, the AUC value of logistic regression was 0.740, indicating overall good performance; Light Gradient Boosting Machine and neural network followed closely; the F1 score of KNN was 0.833; the precision of Light Gradient Boosting Machine was 0.913. The models showed differences across various metrics. Detailed results are shown in Table 8 and Figure 5.

**Table 8:** The external validation results of the seven risk prediction models.

Models	Accuracy	Precision	Recall	F1	AUC
Logistic	0.711	0.909	0.735	0.813	0.740
KNN	0.735	0.903	0.773	0.833	0.684
LightGBM	0.714	0.913	0.735	0.815	0.736
ANN	0.716	0.910	0.741	0.817	0.736
RF	0.703	0.910	0.724	0.806	0.689
SVM	0.701	0.908	0.724	0.805	0.728
XGBoost	0.637	0.925	0.626	0.746	0.714

### 3.7 Construction of the nomogram

**Figure 6:**
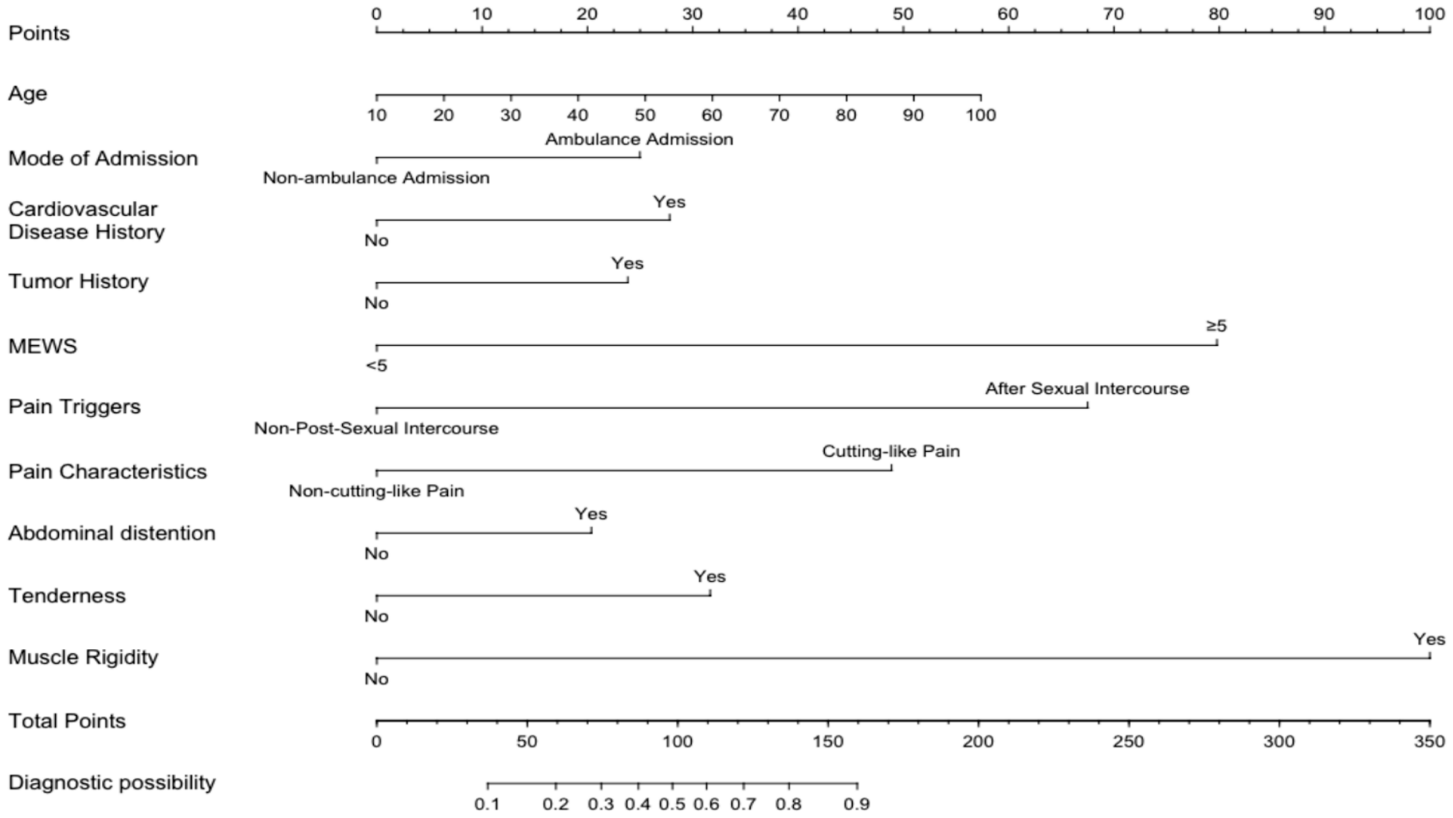
High-risk nomogram for patients with non-traumatic acute abdominal pain.

External validation results indicate that the logistic regression model demonstrates robust generalization capabilities and strong extrapolative potential. To enhance usability in clinical practice, this study developed a high-risk nomogram model for non-traumatic acute abdominal pain patients, utilizing multivariate analysis results and incorporating the 10 identified independent risk factors, as illustrated in Figure 6. Each variable is assigned a specific score, which is summed to produce a total score. The total score corresponds to a value on the Diagnostic Possibility axis, representing the predicted probability of a high-risk outcome for non-traumatic acute abdominal pain patients. Example: A 70-year-old patient with non-traumatic acute abdominal pain (approximately 38 points), admitted by ambulance (approximately 25 points), with a MEWS score ≥5 (approximately 80 points), and all other options scored zero (0 points), would have a total score of 143 points, corresponding to an approximately 82% probability of a high-risk clinical outcome.

**Figure 7:**
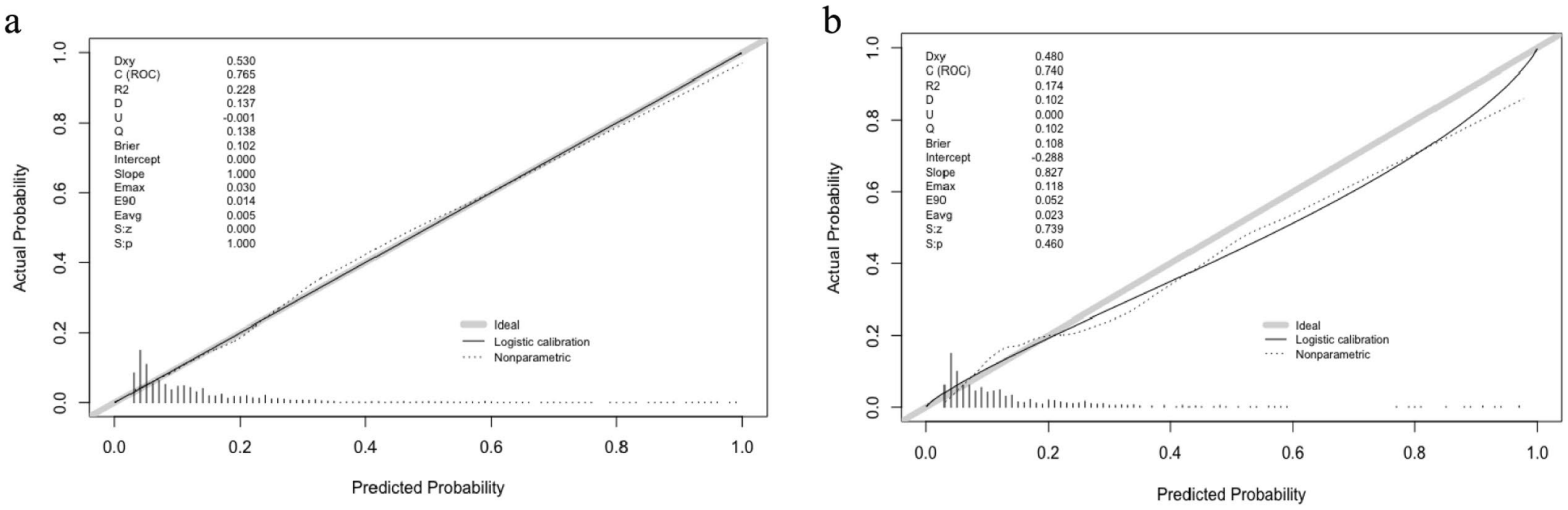
Calibration curve of the logistic regression model. **(a)** Training group. **(b)** Validation group.

The correction curves for both the training and validation models closely approach the ideal curve. The training group has a Brier score of 0.102, Hosmer-Lemeshow (H-L) test = 17.808, *p* = 0.037; the validation group has a Brier score of 0.108, H-L test = 11.365, *p* = 0.252, indicating that the model has good calibration. See Figure 7 for details.

**Figure 8:**
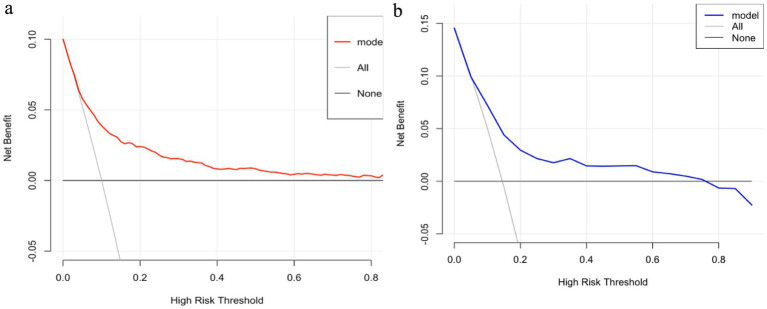
DCA curve of the logistic regression model. **(a)** Training group. **(b)** Validation group.

This study assesses the clinical utility of the logistic regression model by utilizing decision analysis curves. On the clinical decision curve, the horizontal solid line labeled “None” signifies that all samples are negative, with no intervention conducted, leading to a clinical benefit of zero. Conversely, the “All” line shows that all samples are positive and receive interventions; however, clinical benefit ceases when the probability threshold exceeds 10% in the training group and 12% in the validation group. Figure 8 demonstrate that this predictive model extends the benefit threshold.

## 4 Discussion

### 4.1 Current status of high-risk patients with non-traumatic acute abdominal pain

Due to the complex and diverse etiologies of non-traumatic acute abdominal pain and the limited duration of triage (24, 25), under-triage frequently occurs. However, rapid and accurate triage is crucial for reducing the morbidity and mortality of patients with non-traumatic acute abdominal pain (26). Therefore, how to identify high-risk patients with non-traumatic acute abdominal pain early, quickly, and accurately has become a key issue that emergency pre-examination triage personnel urgently need to address from the perspective of holistic health (11).

The results of this study showed that there were 358 high-risk patients with non-traumatic acute abdominal pain, accounting for 14.49%. This is lower than previous studies (12), which may be primarily due to differences in the definition of “high-risk abdominal pain.” Gan et al. (12) defined high-risk abdominal pain patients as those who had resuscitation records, presented with critical values, required emergency surgery, or died within 24 h of emergency department admission. This definition emphasizes the severity and urgency of the condition and provides more precise guidance for the identification and management of high-risk patients in clinical practice. However, this definition may overly focus on patients with rapidly deteriorating conditions while overlooking those with equally severe but more slowly progressing illnesses. This study adopted the definition of high-risk patients proposed in the OPTIMA study. By analyzing the clinical outcomes of patients in the high-risk group, it was found that this definition was effectively validated in the present study and demonstrated good applicability.

### 4.2 Independent risk factors for non-traumatic acute abdominal pain

This study identified a series of independent risk factors for high-risk patients with non-traumatic acute abdominal pain through analytical methods such as LASSO regression and multivariate logistic regression, including age, mode of admission, history of heart disease, and history of tumors.

Compared with previous studies, the conclusion that age is a risk factor is consistent (27, 28). Older patients with non-traumatic acute abdominal pain often have multiple comorbidities, atypical symptoms and signs, loss of independence, or fear of facing hospitals and death, leading to delayed medical visits, more complicated conditions, and increased severity, thereby facing greater risks. The mode of hospital admission was also identified as an independent risk factor, which may suggest that different admission pathways reflect variations in the severity and urgency of the disease. Patients who are self-admitted may not have paid sufficient attention to their symptoms at the initial stage, resulting in delayed and worsened conditions. Patients with a history of heart disease (OR = 2.705) and a history of tumors (OR = 2.347) have a significantly increased risk of developing non-traumatic acute abdominal pain, possibly due to the common presence of chronic inflammation or vascular abnormalities in these two groups, which can easily trigger ischemic bowel disease or tumor rupture. Keskpaik et al. (29) conducted a retrospective study on emergency department patients with abdominal pain as the chief complaint and found that occult myocardial injury (high-sensitivity cardiac troponin T > 14 ng/L) was an independent risk factor for adverse outcomes. This suggests that clinicians should be more vigilant in patients with abdominal pain and a history of heart disease, and initiate appropriate screening and management strategies early, such as electrocardiogram and high-sensitivity cardiac troponin testing. A MEWS score ≥ 5 (OR = 17.380, 95% CI 5.631–53.62) is an independent risk factor for patients with non-traumatic acute abdominal pain. A study by Chen et al. (30) found that the MEWS score is an independent risk factor for 30-day mortality in emergency department patients, further confirming the central role of MEWS in the triage and management of acute abdominal pain. It is recommended that clinicians promptly perform further examinations and interventions for patients with abnormal vital signs (MEWS ≥ 5).

In this study, there were 33 female patients with abdominal pain caused by postcoital abdominal pain, among whom 16 cases (48.48%) experienced high-risk clinical outcomes. One study (31) indicated that more than half (57.1%) of patients with corpus luteum rupture accompanied by hemoperitoneum had engaged in sexual intercourse prior to the onset of pain. Sexual activity may induce ovarian cyst rupture, corpus luteum rupture, and fallopian tube torsion through mechanical stimulation or changes in intra-abdominal pressure. Siddiqui et al. (32) recommended that all women of reproductive age presenting with acute abdominal pain should undergo a comprehensive medical history assessment, including the date of the last menstrual period, use of contraceptives, sexual activity, and other relevant gynecological factors. In clinical practice, the completeness and accuracy of gynecological history documentation can be improved through standardized assessment protocols and electronic records of pregnancy status. In addition, knife-like pain, tenderness, and muscle guarding, as typical symptoms, reflect the pathophysiological process of peritoneal irritation and indicate the need to be alert for gastrointestinal perforation or intra-abdominal infection. Notably, among all samples, 19 patients with non-traumatic acute abdominal pain tested positive for muscle tension position, of whom 18 patients had high-risk clinical outcomes, accounting for 94.74%. These three variables are also key factors in the early risk stratification method for acute abdominal pain developed by Wang et al. (13). Although the significance of abdominal distension and fullness (OR = 2.073) is lower than that of other factors, as a common manifestation of functional intestinal obstruction, it may serve as a critical clue for early identification of high-risk cases in primary healthcare settings. In clinical practice, healthcare personnel should pay close attention to patients with the above risk factors and conduct more comprehensive assessments, which will help in the early identification of high-risk patients, timely medical intervention, and improved patient outcomes.

### 4.3 Performance comparison of seven risk prediction models

With the continuous advancement of computer science and artificial intelligence technologies, their integration with the medical field has further promoted the ongoing development of medical informatization. As a key branch of artificial intelligence, machine learning can integrate heterogeneous and cross-domain data and deeply analyze patient information through computational techniques, thereby enabling the establishment of predictive models. The diagnostic and predictive capabilities of machine learning have been fully validated in multiple fields (33, 34). In this study, data and other observational indicators were prospectively collected under emergency triage conditions, and high-risk early warning models for patients with non-traumatic acute abdominal pain were constructed based on seven algorithms. Precision represents the proportion of samples predicted as high-risk abdominal pain that are indeed patients with high-risk non-traumatic acute abdominal pain, while recall reflects the proportion of all true high-risk non-traumatic acute abdominal pain patients that are successfully identified by the model. Although accuracy and precision share certain similarities in their descriptions, they emphasize different aspects in model performance evaluation: accuracy focuses on the overall correctness of the model’s predictions, taking into account both high-risk and non-high-risk patients, whereas precision concentrates on the proportion of actual high-risk patients within the group predicted as high-risk. To overcome the limitations of single-dimensional metrics, this study further introduces the F1 score and AUC value to establish a multidimensional comprehensive evaluation system, providing a more thorough and objective assessment of the model’s predictive performance. The KNN model achieved the highest prediction accuracy, followed by the RF model. In terms of precision, all models demonstrated values exceeding 0.9, with the RF (0.942) and XGBoost (0.933) models performing particularly well, indicating that these two models possess strong classification capabilities in accurately distinguishing high-risk from non–high-risk patients with non-traumatic acute abdominal pain. The recall rate of the KNN model was 0.802, suggesting that it has high accuracy in identifying high-risk patients and can more effectively detect positive samples. All models achieved an AUC value >0.7, demonstrating strong predictive ability, among which the random forest model (AUC = 0.786) exhibited the best overall predictive performance. Gan et al. (12) included seven risk factors related to non-traumatic acute abdominal pain and constructed predictive models using eight machine learning algorithms. They found that the predictive model based on artificial neural networks performed best, with an AUC value of 0.983, which was higher than that of this study. It is worth noting that their study was retrospective, leading to limitations in feature variables and data collection; moreover, the study did not conduct external validation to assess the extrapolation and generalizability of the model. Given that the random forest model in this study demonstrated the best overall predictive performance, it may be prioritized in clinical practice for high-risk screening of patients with non-traumatic acute abdominal pain, to assist in triage decision-making, improve the accuracy of early assessment, and reduce under-triage. At the same time, healthcare personnel should fully recognize that although different models have value, each has its own characteristics and can be flexibly applied according to actual conditions. For example, in resource-limited settings requiring rapid assessment, the results of the logistic regression model may be referenced. When facing complex medical conditions that require comprehensive analysis of multiple potential factors, the multi-feature handling capability of the random forest model may provide a more holistic perspective. For future research directions, on the one hand, the scope of risk factors can be further expanded, not limited to those collected in the pre-triage environment, but also including indicators such as blood test results and imaging characteristics, in order to improve the accuracy and comprehensiveness of model prediction and to explore in depth the impact of different indicator combinations on model performance. On the other hand, multicenter studies can be conducted to increase the sample size and verify the stability and applicability of different models in different regions and medical settings, providing a more solid foundation for the widespread implementation of the models.

### 4.4 Model generalization and interpretability

Temporal validation closely mirrors the dynamic nature of clinical practice, where patient data is continuously updated and models must adapt to evolving patterns of disease and healthcare utilization. By training the model on data from May to October 2024 and validating it on data from November to December 2024, we aimed to assess the model’s ability to generalize to future patient cohorts, providing a more realistic evaluation of its performance in a clinical setting. This method allows for a more accurate assessment of the model’s practical utility, particularly in environments where healthcare data is subject to temporal changes and seasonal variations. This result may be attributed to the relatively simple structure of the logistic regression model, its effective capture of linear relationships, and its good adaptability to data in common medical scenarios. Although the remaining six models performed slightly less well in this external validation, they show potential in mining data features from different dimensions, and clinicians may flexibly choose among them based on actual clinical needs.

Interpretability of clinical prediction models is crucial for clinical decision-making (35), referring to the extent to which users can understand and explain the prediction process and results of the model. A nomogram is a visual representation of a regression model, which transforms complex mathematical models presented in statistical analyses into simple and visual graphics. Users can directly read the predicted probability values of outcomes from the graph to assist in the prediction of clinical events, offering high interpretability and transparency. In this study, a high-risk nomogram model for patients with non-traumatic acute abdominal pain was constructed based on LASSO regression and logistic regression, enabling accurate prediction and risk stratification of high-risk patients. Users can input 10 clinical parameters of a given patient into the nomogram, sum the corresponding scores, and draw a vertical line at the total score. The intersection with the risk axis at the bottom indicates the patient’s risk level. This method is simple and easy to operate and can assist in clinical decision-making. Future research may involve multidisciplinary collaboration to establish cross-disciplinary learning platforms, promoting the integration of knowledge from nursing, computer science, and other disciplines.

### 4.5 Advantages and limitations

The high-risk early warning model for patients with non-traumatic acute abdominal pain constructed in this study provides an innovative solution for emergency triage nursing practice, achieving significant breakthroughs in the following three aspects: firstly, through the design of an information-based implementation pathway, 32 clinically accessible parameters can be obtained in real time during triage assessment, overcoming the limitations of previous models that relied on laboratory-confirmed biomarkers and ensuring the model’s immediate applicability during the triage window. Additionally, a temporal validation framework was used to systematically compare seven machine learning algorithms. Empirical evidence demonstrated that the random forest model had the best discriminative performance, while the logistic regression model exhibited stronger generalizability, providing an evidence-based foundation for selecting scenario-specific models in clinical practice. Moreover, for the first time, a history of tumors and sexual activity were confirmed as independent risk factors for patients with non-traumatic acute abdominal pain, expanding the traditional risk assessment framework and suggesting that the above factors should be incorporated into the triage assessment system for non-traumatic acute abdominal pain.

We acknowledge several limitations in this study. Firstly, the research data were obtained from three tertiary hospitals in the Yangtze River Delta region of China, and the level of medical resource allocation may affect the applicability of the model in primary healthcare institutions. Secondly, our study has several limitations related to variable selection. To maintain practicality for triage, we did not include laboratory indicators (e.g., inflammatory markers), which may have omitted some predictive factors. Additionally, although “duration of pain” was significant in univariate analysis, it was not selected as an independent predictor by the LASSO regression and multivariate analysis, hence its exclusion from the final model. Furthermore, data on concurrent medications (such as anticoagulants, which may increase the risk of hemorrhage) were not collected, as they were not widely supported as standard predictors in the literature we reviewed for variable selection; this could represent an important omitted variable. Nevertheless, the exclusion of laboratory parameters aligns with our goal of creating a pre-assessment tool, but the latter two points highlight potential constraints in the model’s comprehensiveness. Finally, temporal validation may introduce seasonal bias, as patient demographics, disease prevalence (e.g., infectious gastroenteritis), and healthcare-seeking behavior can vary significantly across seasons and months. While temporal validation provides valuable insights into the model’s future performance, it may not fully capture the model’ s inherent performance as effectively as random splitting with cross-validation. Future studies may consider incorporating additional validation strategies to mitigate these potential biases and provide a more comprehensive evaluation of the model’s robustness and applicability across different patient populations and time periods.

## 5 Conclusion

This study, through a prospective multicenter study design, developed and validated a high-risk early warning model for non-traumatic acute abdominal pain patients in emergency triage settings. Systematic analysis based on a temporal validation framework demonstrated that the model system integrating 10 clinical indicators, including age, mode of admission, and history of heart disease, possesses robust predictive performance. Among the models, the random forest model exhibited the best discriminative ability, while the logistic regression model achieved optimal clinical applicability and generalization performance through visualization using a nomogram. The study innovatively identified new independent risk factors such as sexual activity-related triggers, providing empirical evidence for improving the assessment dimensions of non-traumatic acute abdominal pain triage. This model system can be directly embedded into electronic triage systems, enabling triage nurses to rapidly identify high-risk non-traumatic acute abdominal pain patients during the initial assessment phase through history taking and physical examination alone, demonstrating significant potential for clinical translation. Future research should validate the model’s generalizability across diverse medical settings and explore dynamic risk warning mechanisms to further enhance the efficiency of emergency resource allocation.

## Data Availability

The raw data supporting the conclusions of this article will be made available by the authors, without undue reservation.
